# Adult Children’s Education and Older Mothers’ Health: Are Offspring’s Problems Mediators or Moderators?

**DOI:** 10.1093/geroni/igab046.812

**Published:** 2021-12-17

**Authors:** Robert Frase, Shawn Bauldry, J Jill Suitor, Megan Gilligan

**Affiliations:** 1 Purdue University, West Lafayette, Indiana, United States; 2 Purdue University, Purdue University, Indiana, United States; 3 Iowa State University, Iowa State University, Iowa, United States

## Abstract

Despite the growing body of literature documenting positive effects of adult children’s education on older mothers’ health outcomes there is limited research exploring the mechanisms that underlie and influence this relationship. This lack of knowledge limits our understanding of how or under what conditions older mothers benefit from their offspring’s resources. In this paper, we draw from theories of the life course, cumulative inequality, and the social foreground to explore how adult children’s problems (physical and emotional, personal and financial, and deviant behaviors) mediate and moderate the effect of adult children’s education on older mothers’ self-rated health and depressive symptoms. To address this question we use data collected from 420 mothers aged 75-85 reporting on their 1,514 adult children, as part of the Within Family Differences Study. Theoretically, this project adds to existing scholarship on intergenerational support in later-life families by identifying the conditions under which adult children’s resources improve parents’ well-being. Preliminary findings reveal that less educated adult children experience more problems, which in turn, negatively impact mothers’ health. Additionally, when adult children experience problems in their own lives, mothers receive less care and financial support from their offspring, even from those who are well-educated and would otherwise have been expected to have shared resources. The findings will have implications for practice by increasing health care providers’ awareness that older parents may be at risk for unmet needs for care even when adult children have resources that would have been expected to serve as a safety net.

